# Supplementary material for: Bioinformatic Identification of CRISPR–Cas Systems in Leptospira Genus: An Update on Their Distribution Across 77 Species
Source: Pathogens. 2025 Oct 16;14(10):1044. doi: 10.3390/pathogens14101044 (PMC12567085; doi:10.3390/pathogens14101044)

MDPI
Supplementary Material. Table S1.

pathogens

Table S1. The table summarizes the number of direct repeats, spacer sequences, and bacteriophages identified (based on gene fragments, score, e-value, identity [%]), in the 19 *Leptospira* species with a complete CRISPR-Cas system (Cas proteins, direct repeats, and spacer sequences). It also includes the original hosts of the spacer sequences und unidentified spacers. Color coding indicates subgroup: green for pathogenic P1, blue for pathogenic P2, yellow for saprophytic S1, and red for saprophytic S2.

Name	Subgroup	Number of Unique Direct Repetitions	Number of Spacers	Host	Phage	Score	E-Value	Identity	Unidentified Spacers
L. adleri	P1	19	29	Arthrobacter	phage WaddleDee	30.2	4.6	15/15 (100%)	5
					phage Chubster	32.2	2.9	19/20 (95%)	
					phage SJReid	32.2	1.3	16/16 (100%)	
					phage Rockabye	30.2	4.8	15/15 (100%)	
					phage Bouchard	32.2	1.3	16/16 (100%)	
				Curtobacterium	phage Prophecy	36.2	0.056	18/18 (100%)	
				Gordonia	phage Malisha	32.2	1.6	16/16 (100%)	
					phage Sidious	30.2	5.4	15/15 (100%)	
					phage GMA2	32.2	1.3	16/16 (100%)	
				Microbacterium	phage Naby	32.2	1	16/16 (100%)	
					phage Yuma	30.2	4.8	15/15 (100%)	
					phage Platte	30.2	5.1	18/19 (94%)	
				Mycobacterium	phage Iwokeuplikedis	30.2	6.4	15/15 (100%)	
					phage Piro94	32.2	1.3	16/16 (100%)	
					phage Onyinye	32.2	1.4	19/20 (95%)	

				Propionibacterium	phage P101A	34.2	0.34	17/17 (100%)	
				Rhodococcus	phage Trina	32.2	1.4	16/16 (100%)	
					phage NiceHouse	30.2	4.6	18/19 (94%)	
					phage Weasels2	34.2	0.33	17/17 (100%)	
				Streptomyces	phage SeresaTree	36.2	0.078	18/18 (100%)	
					phage Bmoc	32.2	2.1	16/16 (100%)	
					phage Tomas	40.1	0.009	20/20 (1000%)	
					phage WhereRU	30.2	5.1	15/15 (100%)	
					phage Kenrey	34.2	0.34	17/17 (100%)	
L. alexanderi	P1	29	57	Arthrobacter	phage Trustiboi	30.2	4.3	15/15 (100%)	15
					phage Anekin	32.2	1.1	16/16 (100%)	
					phage Phroglets	30.2	4.3	15/15 (100%)	
					phage Yeezus	30.2	4	15/15 (100%)	
					phage Sporto	30.2	3.7	15/15 (100%)	
					phage Zartrosa	30.2	4.3	15/15 (100%)	
					phage Leathea	30.2	6.2	18/19 (94%)	
					phage Maureen	32.2	1	16/16 (100%)	
					phage Jinkies	30.2	5.6	15/15 (100%)	
				Gordonia	phage MoonFlower	30.2	4	15/15 (100%)	
					phage Warrior24	34.2	0.27	20/21 (95%)	
					phage Volt	32.2	1.1	16/16 (100%)	
					phage Rickmore	32.2	1	16/16 (100%)	

					phage Lollipop1437	30.2	5.6	15/15 (100%)	
					phage Keelan	30.2	5.6	15/15 (100%)	
					phage Twinkle	30.2	5.6	15/15 (100%)	
					phage NHagos	30.2	3.7	15/15 (100%)	
					phage Mollymur	32.2	1.2	16/16 (100%)	
					phage Doggs	30.2	4.5	15/15 (100%)	
				Microbacterium	phage Zepp	30.2	4	15/15 (100%)	
					phage Nebulous	30.2	4.3	15/15 (100%)	
					phage WestPM	30.2	4.3	15/15 (100%)	
					phage TrippleS	32.2	1.2	16/16 (100%)	
					phage Teng	36.2	0.069	18/18 (100%)	
					phage SilentWarrior	30.2	4.3	15/15 (100%)	
				Mycobacterium	phage Zizzle	30.2	4.6	15/15 (100%)	
					phage Indlulamithi	30.2	4.3	15/15 (100%)	
					phage Cracklewink	30.2	4.3	15/15 (100%)	
					phage Whabigail7	32.2	1.1	16/16 (100%)	
					phage Indlulamithi	30.2	5.9	18/19 (94%)	
					phage Zelink	32.2	1.2	16/16 (100%)	
					phage BabyDotz	32.2	1.4	16/16 (100%)	
					phage Youngblood	32.2	1.4	16/16 (100%)	
					phage Thumb	30.2	2.4	15/15 (100%)	
					phage Kumao	32.2	1.2	16/16 (100%)	

				Rhodococcus	phage NiceHouse	34.2	0.29	17/17 (100%)	
					phage Peregrin	32,2	1.2	19/20 (95%)	
				Rothia	phage Spartoi	30.2	5.4	15/15 (100%)	
				Streptomyces	phage Thiqqums	30.2	4.3	18/19 (94%)	
					phage Beleetus	30.2	4.3	15/15 (100%)	
					phage StarPlatinum	30.2	4	15/15 (100%)	
					phage Jada	32.2	1.1	16/16 (100%)	
L. alstonii	P1	13	21	Arthrobacter	phage GantcherGoblin	30.2	4.8	15/15 (100%)	6
					phage Tallboi	34.2	0.31	17/17 (100%)	
					phage Vopal	30.2	5.1	15/15 (100%)	
				Gordonia	phage Syleon	32.2	2.4	16/16 (100%)	
					phage Morrissey	32.2	1.4	16/16 (100%)	
					phage Wheezy	32.2	1.3	16/16 (100%)	
					phage Yikes	30.2	4.8	18/19 (94%)	
				Microbacterium	phage Xitlalli	32.2	2.3	16/16 (100%)	
				Mycobacterium	phage Reindeer	32.2	2	16/16 (100%)	
					phage TootsiePop	30.2	4.8	15/15 (100%)	
					phage Bromden	30.2	5.4	15/15 (100%)	
				Rhodococcus	phage Poco6	30.2	8.3	15/15 (100%)	
				Streptomyces	phage MeganTheeKilla	34.2	0.33	17/17 (100%)	
					phage Targaryen	34.2	0.33	17/17 (100%)	
					phage Marsus	30.2	5.1	15/15 (100%)	

L. borgpetersenii	P1	11	18	Arthrobacter	phage JKerns	36.2	0.13	21/22 (95%)	7
					phage Wollypog	32.2	1.9	16/16 (100%)	
					phage Patbob	30.2	4.3	15/15 (100%)	
					phage WaddleDee	34.2	0.27	17/17 (100%)	
				Gordonia	phage BobRoss2024	32.2	2.2	16/16 (100%)	
					phage Shatter	30.2	4.6	15/15 (100%)	
					phage Turuncu	30.2	4.3	15/15 (100%)	
				Microbacterium	phage Count	30.2	4.3	15/15 (100%)	
				Mycobacterium	phage Weirdo19	30.2	9.4	15/15 (100%)	
					phage Onyinye	30.2	4.3	15/15 (100%)	
				Rhodococcus	phage DocB7	30.2	4.6	15/15 (100%)	
L. gorisiae	P1	7	28	Arthrobacter	phage Xenomorph	28.2	9.5	14/14 (100%)	17
					phage Phrampa	32.2	0.75	16/16 (100%)	
				Gordonia	phage Yeshua	32.2	1.2	19/20 (95%)	
					phage Phendrix	32.2	1.1	16/16 (100%)	
					phage Rabbitrun	30.2	4.3	15/15 (100%)	
				Mycobacterium	phage Zebo	34.2	0.27	17/17 (100%)	
					phage ZygoTaiga	28.2	9.5	14/14 (100%)	
				Rhodococcus	phage Peregrin	30.2	4.3	15/15 (100%)	
				Streptomyces	phage Bing	34.2	0.27	17/17 (100%)	
					phage Yaboi	30.2	4.3	15/15 (100%)	
					phage Beleetus	28.2	9.5	14/14 (100%)	

L. interrogans	P1	22	13	Curtobacterium	phage Commonplace	30.2	5.6	15/15 (100%)	5
				Gordonia	phage GMA3	34.2	0.34	17/17 (100%)	
				Microbacterium	phage PauloDiaboli	30.2	6.4	15/15 (100%)	
					phage Zahlia	30.2	8.3	15/15 (100%)	
				Streptomyces	phage Sros11	30.2	7.5	15/15 (100%)	
					phage Tomas	28.2	9.5	14/14 (100%)	
					phage Phredrick	32.2	1.2	16/16 (100%)	
					phage Jada	32.2	1.6	16/16 (100%)	
L. kirschneri	P1	37	29	Arthrobacter	phage Sunny4976	30.2	7.8	15/15 (100%)	8
					phage Inked	30.2	4.6	18/19 (94%)	
					phage Urla	30.2	5.9	15/15 (100%)	
					phage LadyJasley	32.2	1.4	16/16 (100%)	
				Gordonia	phage PatrickStar	30.2	7.2	15/15 (100%)	
					phage GMA2	30.2	8.6	15/15 (100%)	
					phage MissRona	32.2	2.8	16/16 (100%)	
					phage Thimann	32.2	1.2	16/16 (100%)	
					phage Wojtek	30.2	5.6	15/15 (100%)	
				Microbacterium	phage Rasputia	32.2	2	19/20 (95%)	
					phage Triscuit	30.2	5.4	15/15 (100%)	
				Mycobacterium	phage Chargerpower	32.2	1.2	16/16 (100%)	
				Rhodococcus	phage Peregrin	34.2	0.46	17/17 (100%)	
					phage NiceHouse	30.2	7.2	15/15 (100%)	

					phage RegnumDei	34.2	0.33	17/17 (100%)	
				Streptomyces	phage TunaTartare	30.2	7.2	15/15 (100%)	
					phage Blueeyedbeauty	30.2	8	15/15 (100%)	
					phage Cursive	30.2	8	18/19 (94%)	
					phage Faust	32.2	1.3	19/20 (95%)	
					phage Jada	32.2	0.74	16/16 (100%)	
					phage Wofford	30.2	6.7	15/15 (100%)	
L. kmetyi	P1	11	28	Arthrobacter	phage Rizwana	34.2	0.33	17/17 (100%)	9
					phage WaddleDee	32.2	1.3	16/16 (100%)	
					phage KNG13	30.2	5.4	15/15 (100%)	
				Brevibacterium	phage Lukepolites	32.2	1.3	16/16 (100%)	
				Curtobacterium	phage Sweetclover	30.2	5.1	21/23 (91%)	
				Gordonia	phage Lilbeanie	32.2	1.6	16/16 (100%)	
					phage Mcklovin	30.2	4.8	15/15 (100%)	
					phage BluerMoon	32.2	1.2	16/16 (100%)	
				Microbacterium	phage Pumpernickel	34.2	0.36	17/17 (100%)	
				Mycobacterium	phage Madruga	30.2	4.8	15/15 (100%)	
					phage Tourach	32.2	1.4	16/16 (100%)	
					phage Kropertea	34.2	0.31	17/17 (100%)	
					phage QueenHazel	30.2	5.1	15/15 (100%)	
					phage WeiHuaDA	30.2	4.8	15/15 (100%)	
				Rhodococcus	phage Peregrin	30.2	5.1	15/15 (100%)	

				Streptomyces	phage Wilkos	30.2	4.8	18/19 (94%)	
					phage Watermoore	30.2	5.1	15/15 (100%)	
					phage Beuffert	32.2	1.3	16/16 (100%)	
					phage South40	32.2	1.3	16/16 (100%)	
L. mayottensis	P1	20	47	Arthrobacter	phage Nason	30.2	4.3	15/15 (100%)	18
					phage Arzan	32.2	1.1	19/20 (95%)	
					phage RedFox	32.2	1.4	16/16 (100%)	
					phage ScienceWizSam	30.2	4.5	15/15 (100%)	
				Corynebacterium	phage MicyPS	36.2	0.069	18/18 (100%)	
				Gordonia	phage Twin	30.2	6.7	15/15 (100%)	
					phage Yeshua	32.2	1.4	19/20 (95%)	
					phage Tredge	32.2	1.1	16/16 (100%)	
					phage Squiddly	32.2	1.1	16/16 (100%)	
					phage GordTnk2	32.2	1.1	19/20 (95%)	
				Microbacterium	phage Zanella	34.2	0.27	17/17 (100%)	
					phage TwoBits	36.2	0.069	18/18 (100%)	
					phage Tyrumbra	30.2	4.3	15/15 (100%)	
					phage ValentiniPuff	30.2	4.3	15/15 (100%)	
				Mycobacterium	phage YoSam321	32.2	1.1	16/16 (100%)	
					phage Antsirabe	30.2	4.3	15/15 (100%)	
					phage SuperSonics	30.2	4.3	15/15 (100%)	
					phage DmpstrDiver	32.2	1.1	16/16 (100%)	

					phage Paito	32.2	1.1	16/16 (100%)	
					phage Ruotula	32.2	1.1	16/16 (100%)	
					phage Indlulamithi	30.2	4.3	15/15 (100%)	
				Streptomyces	phage Blueeyedbeauty	30.2	4.3	15/15 (100%)	
					phage TunaTartare	32.2	1.1	16/16 (100%)	
					phage Wakanda	32.2	1.1	16/16 (100%)	
					phage Wipeout	32.2	1.1	19/20 (95%)	
					phage Garnacho	36.2	0.069	18/18 (100%)	
					phage Warpy	30.2	4.3	15/15 (100%)	
					phage Watermoore	30.2	4.3	15/15 (100%)	
					phage Riptide	30.2	4.5	15/15 (100%)	
L. noguchii	P1	28	47	Arthrobacter	phage Bouchard	30.2	4.8	18/19 (94%)	22
					phage Panchaali	30.2	5.1	15/15 (100%)	
					phage KeaneyLin	30.2	4.8	15/15 (100%)	
					phage YoungHarleezy	32.2	1.2	16/16 (100%)	
					phage AWGoat	30.2	5.1	15/15 (100%)	
					phage Talia1610	30.2	5.1	15/15 (100%)	
					phage Gorpy	32.2	1.4	16/16 (100%)	
					phage Westrich	32.2	1.3	16/16 (100%)	
				Curtobacterium	phage Kithara	34.2	0.33	17/17 (100%)	
				Gordonia	phage Zeph	30.2	8.3	15/15 (100%)	
					phage StarStruck	30.2	4.6	15/15 (100%)	

					hage YorkOnyx	32.2	1.3	16/16 (100%)	
					phage Keelan	32.2	1.4	16/16 (100%)	
					phage Vardy	32.2	1.2	16/16 (100%)	
				Microbacterium	phage PauloDiaboli	30.2	7.5	15/15 (100%)	
					phage Tempo	30.2	5.1	15/15 (100%)	
					phage Eleri	32.2	1.2	16/16 (100%)	
					phage Shamu	30.2	5.4	15/15 (100%)	
					phage Zooman	30.2	5.4	15/15 (100%)	
				Rhodococcus	phage Pepy6	32.2	1.3	16/16 (100%)	
				Streptomyces	phage NootNoot	36.2	0.078	21/22 (95%)	
					phage TunaTartare	30.2	5.1	15/15 (100%)	
					phage Tomas	30.2	5.1	15/15 (100%)	
					phage Limpid	30.2	7.5	15/15 (100%)	
					phage Jada	32.2	1.9	16/16 (100%)	
L. santarosai	P1	32	33	Arthrobacter	phage AWGoat	32.2	1.1	16/16 (100%)	9
					phage WonderBoy	34.2	0.34	20/21 (95%)	
					phage Sonali	32.2	1.7	16/16 (100%)	
				Curtobacterium	phage Mantle	32.2	1.2	19/20 (95%)	
				Gordonia	phage Perkunas	30.2	3.5	15/15 (100%)	
					phage Dmitri	32.2	1.8	16/16 (100%)	
					phage Zeph	32.2	1.6	16/16 (100%)	
					phage Denise	30.2	7.8	15/15 (100%)	

					phage Worcestershire	30.2	4.3	15/15 (100%)	
					phage StarStruck	36.2	0.069	18/18 (100%)	
					phage UBSmoodge	30.2	4.5	15/15 (100%)	
				Microbacterium	phage WaterT	30.2	5.1	15/15 (100%)	
				Mycobacterium	phage XFactor	32.2	1.1	16/16 (100%)	
					phage Vincenzo	30.2	5.4	15/15 (100%)	
					Phage MrMagoo	30.2	4	15/15 (100%)	
					phage ThulaThula	30.2	4.3	15/15 (100%)	
					phage Xula	30.2	4.3	15/15 (100%)	
					phage Yecey3	32.2	1	16/16 (100%)	
					phage Zenteno07	32.2	1.3	16/16 (100%)	
				Streptomyces	phage Yara	30.2	3.7	15/15 (100%)	
					phage ZooBear	32.2	1.4	19/20 (95%)	
					phage Yaboi	30.2	5.4	15/15 (100%)	
					phage Bordeaux	30.2	4.3	15/15 (100%)	
					phage WhereRU	30.2	4.3	15/15 (100%)	
L. stimsonii	P1	13	25	Arthrobacter	phage Vulture	30.2	5.4	15/15 (100%)	7
					phage Trustiboi	28.2	9.5	14/14 (100%)	
					phage AinMach	32.2	1.1	16/16 (100%)	
				Gordonia	phage Zareef	30.2	4.3	15/15 (100%)	
				Microbacterium	phage Pumpernickel	32.2	1,2	16/16 (100%)	
					phage TwoBits	30.2	4.3	18/19 (94%)	

				Mycobacterium	phage SuperSonics	28.2	6.3	14/14 (100%)	
					phage Toron	30.2	4.8	15/15 (100%)	
					phage Zaider	32.2	2.9	16/16 (100%)	
					phage Chargerpower	30.2	4.6	15/15 (100%)	
					phage Waleliano	30.2	4.3	15/15 (100%)	
					phage Jabiru	32.2	1,1	16/16 (100%)	
				Streptomyces	phage Targaryen	30.2	1.6	15/15 (100%)	
					phage Phredrick	30.2	6.2	15/15 (100%)	
					phage RosaAsantewaa	34.2	0.43	20/21 (95%)	
					phage Wofford	30.2	4.3	15/15 (100%)	
					phage Westy	30.2	4.3	15/15 (100%)	
					phage Andris	30.2	4.3	15/15 (100%)	
L. weilii	P1	10	38	Arthrobacter	phage LadyJasley	30.2	4.3	15/15 (100%)	17
					phage BlueShadow	30.2	4.3	15/15 (100%)	
				Brevibacterium	phage Cantare	30.2	4.3	15/15 (100%)	
				Corynebacterium	phage PSonyx	30.2	4.3	15/15 (100%)	
				Gordonia	phage RoyalG	34.2	0.67	17/17 (100%)	
					phage Lucky10	34.2	0.27	17/17 (100%)	
					phage Lucky10	34.2	0.27	17/17 (100%)	
					phage SteveFrench	36.2	0.069	18/18 (100%)	
					phage Yakult	30.2	4	15/15 (100%)	
					phage BenoitCattle	30.2	4.3	15/15 (100%)	

					phage Alephilan	30.2	4.3	15/15 (100%)	
					phage ZiggyZoo	36.2	0.065	21/22 (95%)	
					phage Ziko	30.2	4,3	15/15 (100%)	
				Microbacterium	phage Zhafia	30.2	4.3	15/15 (100%)	
				Mycobacterium	phage Fowlmouth	30.2	4.3	15/15 (100%)	
					phage Tortellini	30.2	4.3	15/15 (100%)	
				Rhodococcus	phage Reynauld	30.2	4.8	15/15 (100%)	
					phage RegnumDei	30.2	4,3	15/15 (100%)	
				Streptomyces	phage Success	34.2	0.31	17/17 (100%)	
					phage GiJojo	34.2	0.7	17/17 (100%)	
					phage Enygma	30.2	4.3	18/19 (94%)	
L. fainei	P2	11	31	Arthrobacter	phage Anekin	34.2	0.27	17/17 (100%)	7
					phage StuartMinion	32.2	1.1	16/16 (100%)	
					phage Rockabye	30.2	4.3	15/15 (100%)	
				Corynebacterium	phage MicyPS	30.2	4.3	15/15 (100%)	
				Gordonia	phage Zareef	32.2	1.1	16/16 (100%)	
					phage Lilbeanie	30.2	4.3	15/15 (100%)	
					phage LuckyLeo	34.2	0.27	20/21 (95%)	
					phage TenaciousP	30.2	4.3	15/15 (100%)	
					phage PierreThree	30.2	4.3	15/15 (100%)	
					phage Terapin	32.2	1.1	19/20 (95%)	
					phage TenaciousP	30.2	4.3	15/15 (100%)	

				Microbacterium	phage Burro	32.2	1.2	16/16 (100%)	
				Mycobacterium	phage Predator	30.2	4.3	15/15 (100%)	
					phage Zakhe101	32.2	1.1	16/16 (100%)	
					phage Wildcat	32.2	1.1	16/16 (100%)	
				Streptomyces	phage Vanseggelen	30.2	4	15/15 (100%)	
					phage Tribute	30.2	4.3	15/15 (100%)	
					phage Paradiddles	32.2	1.1	16/16 (100%)	
					phage MulchMansion	30.2	4.3	15/15 (100%)	
					phage Yaboi	32.2	1.2	16/16 (100%)	
					phage Sham	30.2	4.3	15/15 (100%)	
					phage WhereRU	30.2	4.3	15/15 (100%)	
					phage Wipeout	30.2	4.5	15/15 (100%)	
					phage KimJongPhill	32.2	1.2	16/16 (100%)	
L. fletcheri	P2	3	15	Arthrobacter	phage Sunny4976	30.2	4.8	15/15 (100%)	3
					phage Lizalica	34.2	0.31	17/17 (100%)	
				Gordonia	phage RobinSparkles	30.2	4.8	15/15 (100%)	
					phage Phendrix	30.2	4.6	15/15 (100%)	
				Mycobacterium	phage Zilizebeth	30.2	4.8	15/15 (100%)	
					phage TingHuaYa	34.2	0.27	17/17 (100%)	
					phage Badge	34.2	0.33	17/17 (100%)	
				Rhodococcus	phage DocB7	32.2	1.2	16/16 (100%)	
				Streptomyces	phage Kela	34.2	0.36	20/21 (95%)	

					phage Phredrick	36.2	0.078	18/18 (100%)	
					phage Patelgo	30.2	4.8	18/19 (94%)	
					phage pZL12	30.2	4.8	15/15 (100%)	
L. inadai	P2	9	39	Arthrobacter	phage Loretta	41.2	0.003	21/21 (100%)	13
					phage Qui	32.2	1.3	16/16 (100%)	
					phage Piccoletto	30.2	5.1	15/15 (100%)	
					phage Zodiariah	30.2	5.1	15/15 (100%)	
					phage IsHungry	30.2	5.1	15/15 (100%)	
					BillyTP	30.2	5.1	15/15 (100%)	
					phage CastorTray	32.2	0.68	16/16 (100%)	
					phage Altostratus	32.2	0.68	16/16 (100%)	
					phage Panchaali	28.2	9.5	14/14 (100%)	
				Gordonia	phage Sixama	30.2	5.1	15/15 (100%)	
					phage Trax	30.2	5.1	15/15 (100%)	
				Microbacterium	phage WaterT	32.2	0.74	16/16 (100%)	
					phage PermaG	30.2	5.1	15/15 (100%)	
				Mycobacterium	phage Wildcat	28.2	6.3	14/14 (100%)	
					phage FairyPath	28.2	6.3	14/14 (100%)	
					phage Muddy	32.2	1.1	16/16 (100%)	
					phage GenevaB15	30.2	5.1	15/15 (100%)	
					Phage Nazo	32.2	1.3	19/20 (95%)	
					phage MrMiyagi	30.2	5.1	15/15 (100%)	

				Rhodococcus	phage Grayson	30.2	5.1	15/15 (100%)	
					phage Dorin	32.2	1.3	16/16 (100%)	
				Streptomyces	phage WhereRU	30.2	5.1	15/15 (100%)	
					phage Warpy	30.2	5.1	15/15 (100%)	
					phage Araceli	34.2	0.33	17/17 (100%)	
					phage Kardashian	34.2	0.33	17/17 (100%)	
					phage SV1	32.2	1.3	16/16 (100%)	
L. koniambonensis	P2	2	49	Arthrobacter	phage Correa	32.2	1.1	16/16 (100%)	18
					phage Nightmare	30.2	4.3	18/19 (94%)	
					phage Moki	30.2	4.3	15/15 (100%)	
					phage Hestia	32.2	1.1	16/16 (100%)	
					phage SJReid	30.2	4	15/15 (100%)	
					phage Raqqa	36.2	0.069	18/18 (100%)	
					phage Vulpecula	30.2	4.3	15/15 (100%)	
					phage Tophat	30.2	4.3	15/15 (100%)	
				Corynebacterium	phage Bran	30.2	4.3	15/15 (100%)	
					phage Stickynote	30.2	4.3	15/15 (100%)	
				Gordonia	phage Sienna	32.2	1.1	16/16 (100%)	
					phage YungMoney	30.2	4.3	15/15 (100%)	
					phage GMA6	32.2	1.1	16/16 (100%)	
				Microbacterium	phage Floof	34.2	0.27	17/17 (100%)	
					phage TicTac	32.2	1.1	16/16 (100%)	

					phage Zooman	30.2	4.6	15/15 (100%)	
					phage Zeta1847	32.2	1	16/16 (100%)	
				Mycobacterium	phage Willsammy	30.2	4.3	18/19 (94%)	
					phage Superphikiman	30.2	4.3	15/15 (100%)	
					phage Phalaborwa	30.2	4.3	15/15 (100%)	
					phage Indlulamithi	30.2	4.3	15/15 (100%)	
					phage TyDawg	34.2	0.27	17/17 (100%)	
					phage ZenTime222	30.2	4	15/15 (100%)	
					phage Rubeelu	30.2	4.3	15/15 (100%)	
				Rhodococcus	phage Weasels2	30.2	4.3	21/23 (91%)	
					phage RegnumDei	30.2	4.3	18/19 (94%)	
				Streptomyces	phage Westy	36.2	0.069	18/18 (100%)	
					phage Targaryen	30.2	4.3	18/19 (94%)	
					phage Teutsch	36.2	0.069	18/18 (100%)	
					phage MeganTheeKilla	30.2	4.3	15/15 (100%)	
					phage Paradiddles	34.2	0.27	17/17 (100%)	
L. ilyithenensis	S2	2	12	Corynebacterium	phage JeanGrey	32.2	0.61	16/16 (100%)	4
				Curtobacterium	phage Razzleberry	28.2	9.5	17/18 (94%)	
				Gordonia	phage BrutonGaster	32.2	0.88	16/16 (100%)	
					phage Warrior24	34.2	0.17	17/17 (100%)	
				Microbacterium	phage TicTac	30.2	2.9	15/15 (100%)	
					phage Lupine	30.2	2.9	15/15 (100%)	

				Streptomyces	phage Satis	34.2	0.21	17/17 (100%)	
					phage Dubu	30.2	2.9	15/15 (100%)	
L. ryugenii	S2	8	58	Arthrobacter	phage Vulture	30.2	4,3	15/15 (100%)	23
					phage PeggyLeg03	30.2	4,3	15/15 (100%)	
					phage Tuck	34.2	0.27	17/17 (100%)	
					phage Vitus	30.2	4,0	15/15 (100%)	
					phage Idaho	30.2	2.9	15/15 (100%)	
					phage Sunny4976	28.2	9.5	14/14 (100%)	
					phage Anekin	28.2	9.5	14/14 (100%)	
				Gordonia	phage Keelan	32.2	1.1	16/16 (100%)	
					phage Zany	30.2	4,3	18/19 (94%)	
					phage Untouchable	32.2	1.1	16/16 (100%)	
					phage Phomeo	34.2	0.27	17/17 (100%)	
					phage DumpsterDude	30.2	4,3	15/15 (100%)	
					phage TinaLin	30.2	2.7	15/15 (100%)	
				Microbacterium	phage Zooman	30.2	4,3	15/15 (100%)	
					phage TwoBits	30.2	2.7	15/15 (100%)	
				Mycobacterium	phage MarkPhew	30.2	4	15/15 (100%)	
					phage Neighly	32.2	1	16/16 (100%)	
					phage Rando14	30.2	4,3	15/15 (100%)	
					phage Zaria	32.2	1.1	16/16 (100%)	
					phage TootsiePop	34.2	0.27	17/17 (100%)	

					phage Yuna	30.2	4,3	15/15 (100%)	
					phage Anthony	30.2	5.1	18/19 (94%)	
					phage Wanda	30.2	2.9	15/15 (100%)	
				Propionibacterium	phage Pirate	28.2	9.5	14/14 (100%)	
				Rhodococcus	phage Peregrin	32.2	1.1	16/16 (100%)	
					phage Grayson	30.2	4,3	15/15 (100%)	
					phage Whack	30.2	2.9	15/15 (100%)	
				Streptomyces	phage SparkleGoddess	32.2	1.1	16/16 (100%)	
					phage Samy	30.2	4,3	15/15 (100%)	
					phage Wipeout	30.2	4,3	15/15 (100%)	
					phage Paradiddles	32.2	1.1	16/16 (100%)	
					phage Chaewon	30.2	4,0	15/15 (100%)	
					phage Talos	30.2	2.7	15/15 (100%)	
					phage BRock	30.2	2.7	18/19 (94%)	
				Tsukamurella	phage TPA4	30.2	4,3	15/15 (100%)	

Supplementary Material S1. The file shows the detection of orthologous proteins, analysis of three-dimensional structures, and functional domain analysis between species *Leptospira fletcheri* (Cas9)/Streptococcus pyogenes(Cas9), and *Leptospira inadai* (Cas12a)/ Francisella novicida (Cas12a).

Detection of orthologous proteins. The search for orthologous proteins of *Leptospira fletcheri* (Cas9) and *Leptospira inadai* (Cas12a) was performed using the BLAST algorithm in the NCBI database. These proteins are very similar to those found in other species of the *Leptospira* genus. Furthermore, they have a high percentage of identity, genetic relatedness, and coverage with genetically related genera such as *Turnerella* and *Leptonema*.

Leptospira fletcheri (Cas9)				
**Accession**	**Genus/species**	**Cover**	**E-Value**	**Identity**
WP_039933712	*Leptospira inadai*	71%	0.0	66.25%
MFN3603238.1	*Leptonema sp.*	95%	$8e^{-172}$	32.05%
GAB4438665.1	*Turneriella sp.*	97%	$2e^{-156}$	29.68%
Leptospira inadai (Cas12a)				
**Accession**	**Genus**	**Cover**	**E-Value**	**Identity**
WP_108977930	*Leptospira ryugenii*	100%	0.0	66.72%
HRP68199	*Turneriella sp.*	99%	0.0	47.68%

MDPI Supplementary Material. Table S1.

pathogens

Analysis of three-dimensional structures. The three-dimensional structure of the proteins *Leptospira fletcheri* (Cas9), *Leptospira inadai* (Cas12a), *Streptococcus pyogenes* (Cas9), and *Francisella novicida* (Cas12a) was modeled. There are differences between the Cas proteins (Cas9) of the *Leptospira* genus and the proteins of the genera *Streptococcus pyogenes*, and *Francisella novicida* in the amino acid sequence, functional domains, and proteins structure. However, the Cas12a protein of *Leptospira inadai* contains functional domains and structure identical to those found in *Francisella novicida*, which are model species in the study of the CRISPR-Cas system.

Leptospira fletcheri **Cas9**<br>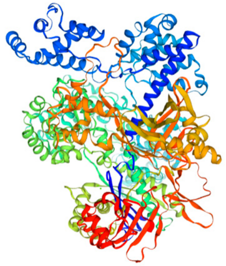	**Streptococcus pyogenes Cas9**<br>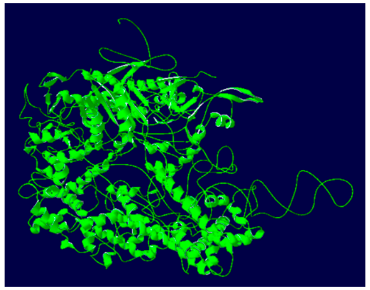	**Structural alignment of *Leptospira fletcheri* Cas9/ Streptococcus pyogenes Cas9**<br>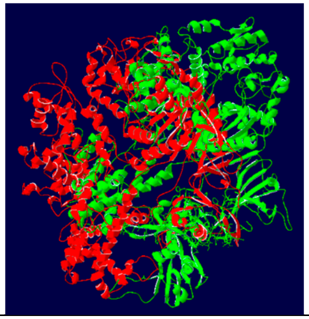
***Leptospira inadai* Cas12a**<br>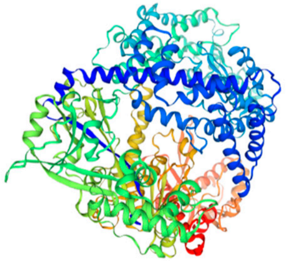	***Francisella novicida* Cas12a**<br>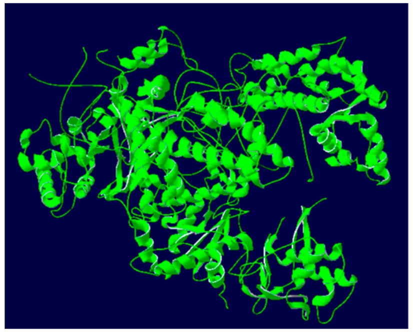	**Structural alignment of *Leptospira inadai* Cas12a / Francisella novicida Cas12a**<br>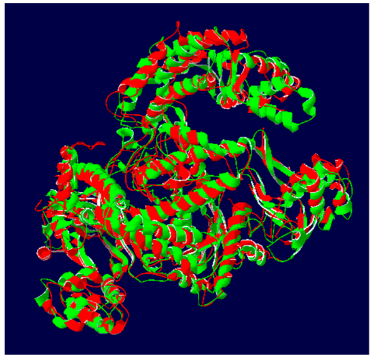

Functional domain analysis. Functional domain analysis in *Leptospira fletcheri* (Cas9), *Leptospira inadai* (Cas12a), *Streptococcus pyogenes* (Cas9), and *Francisella novicida* (Cas12a) were done. Cas12a protein of *Leptospira inadai* contains functional domains identical to those found in *Francisella novicida*, which are model species in the study of the CRISPR-Cas system.

Leptospira fletcheri (Cas9)

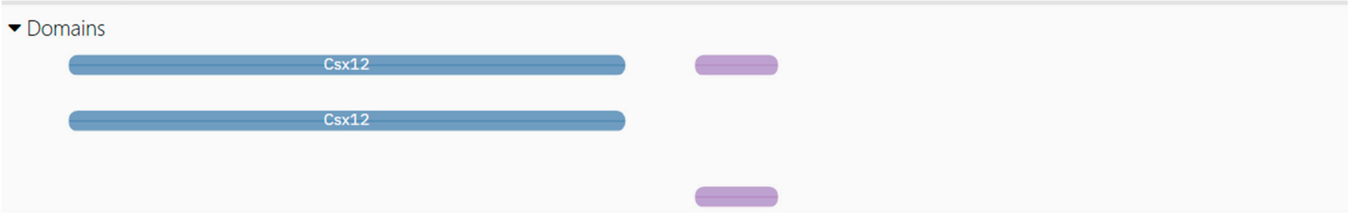

Streptococcus pyogenes (Cas9)

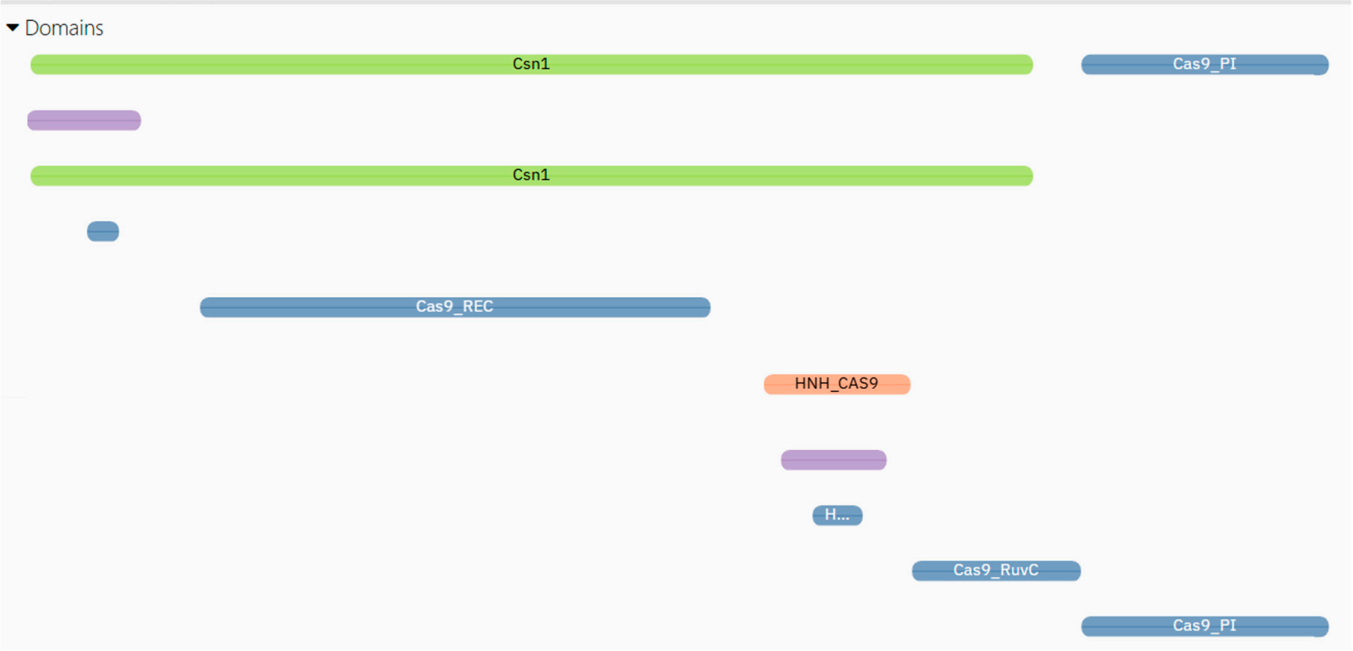

MDPI Supplementary Material. Table S1.
pathogens

Leptospira inadai (Cas12a)

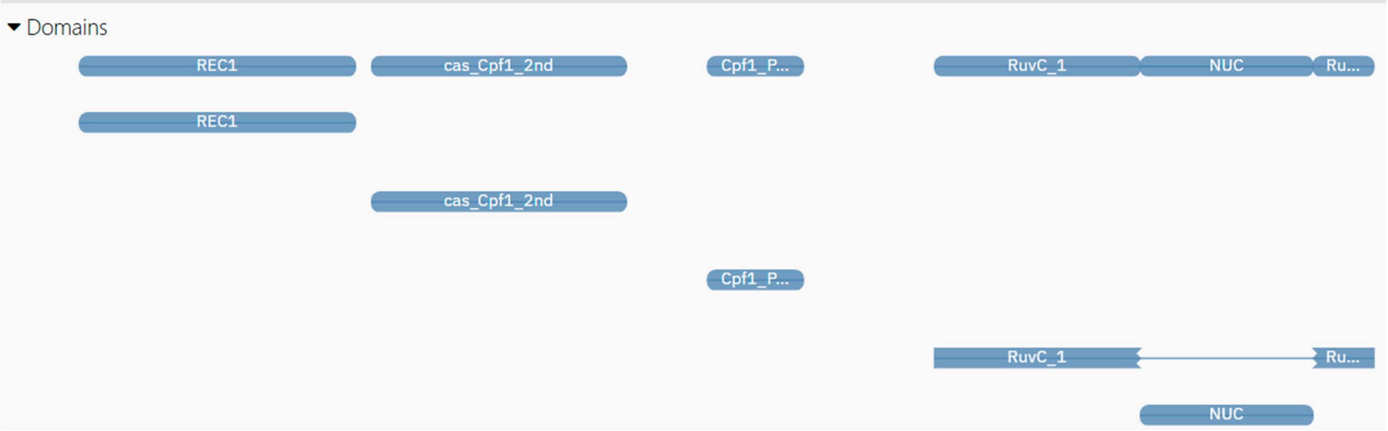

Francisella novicida (Cas12a)

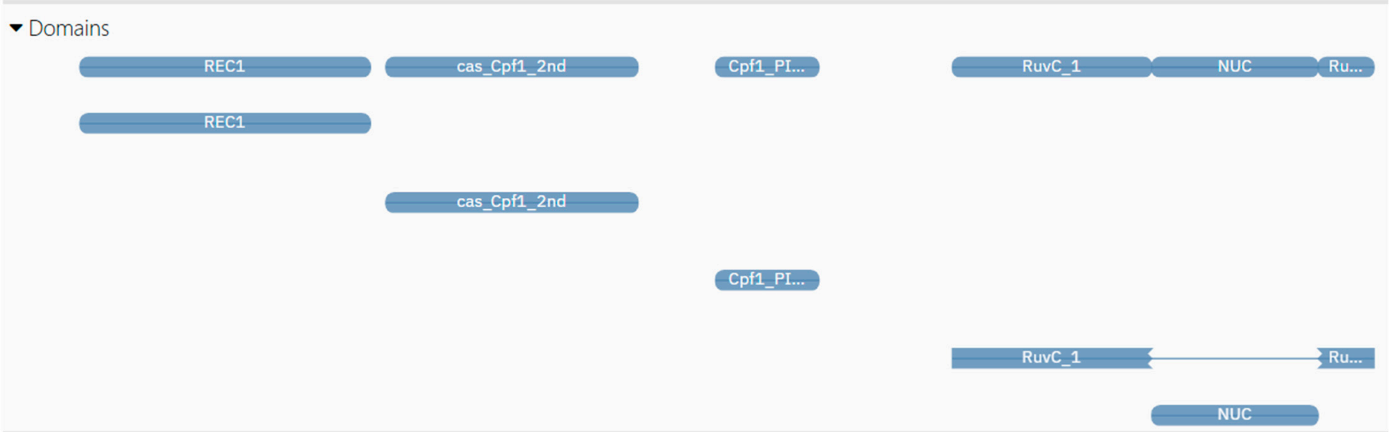

Supplement: Supplementary file 1 [file pathogens-14-01044-s001.zip › pathogens-3885699-supplementary.pdf]
